# Peripheral edema in a diabetic patient on ACE inhibitor: differential diagnosis

**DOI:** 10.1186/1710-1492-8-S1-A18

**Published:** 2012-11-02

**Authors:** Jacques Hébert, Roland R Tremblay

**Affiliations:** 1 Allergy and Immunology, CHUQ/Laval University, Québec City, Canada; 2 Endocrinology, CHUQ/Laval University, Québec City, Canada

## Case history

44 years old man who complained of painful edema of both hands and feet with limited range of motion that interferes with his work (computer). On physical exam, the skin of the hands was thick and tight wax with clear limitation of motion. A pitting edema was seen on both feet. BP was 140/90 and no synovitis was documented. He developed paresthesias on both hands, mostly during the night, and severe carpal tunnel syndrome was documented (proven by EMG). Then, an inability to press the palms together without a gap (called prayer sign) was documented. Past history reveals insulin-dependent diabetes and HBP (on Altace). The complete blood work-up was within normal limits except positive ANA 1:2560, nucleolar pattern with anti-DNA and ENA negative. Because of an inflammatory condition was first suspected, he was placed on prednisone 50 mg daily x2W with no change. The ACE inhibitor was then suspected to be involved in a bradykinin-induced angioedema and was stopped. The patient treated with plasma-derived C1 inhibitor (Berinert) 1500U.(I.V.) and the initial therapeutic response was modest; with further infusion, no significant change was observed. He did not respond either to anti-bradykinin therapy (ICATIBANT) 30 mg (S.C.).

## Conclusion

The whole condition suggests then a rare musculoskeletal complication of long lasting diabetes, called diabetic cheiroarthropathy or diabetic stiff hand syndrome. The underlying cause is mutlifactorial: increased glycosylation of collagen in the skin, decreased collagen degradation, diabetic microangiopathy and possibly neuropathy. What is unique in this case is the involvement of lower limbs with edema, which has never been reported previously. No specific treatment is of this clinical condition is available at present time.

## Summary

We reported a case of peripheral thickening of the skin of both extremities that could be misleading for arthritis or bradykinin-induced angioedema. It is unique in its distribution on upper and lower extremities.

